# Notes and News

**Published:** 1892-10-08

**Authors:** 


### NOTES AND NEWS.

COLLECTED AND COMMUNICATED.

At the new infirmary at Southport, the foundation-stone of which is to be laid shortly by the Mayor, the authorities have pledged themselves to permit of no vivisection within the walls. It is not proposed that the infirmary should be utilized as a medical school.

---

The Hospital Sunday collection at Birmingham is this year to be devoted to the General Hospital, the largest, oldest, and neediest of the Birmingham medical charities. The fund produced nearly £5,000 last year, a sum we will hope to be still further augmented on the present occasion.

---

Already the storm at the Glasgow Royal Infirmary has cleared off, and the authorities have reconsidered their decision to exclude female students from the only clinical instruction open to them in that city. The Infirmary has sufficient resources to allow of separate instruction being given to women without interfering with the men's opportunities. Where this distinct education is possible it must be infinitely preferable to students of both sexes

---

Messrs. Cassell and Company have just brought out a new paper for boys, entitled *Chums* It is issued weekly for the price of one penny, well illustrated, and printed on good paper, and we cannot understand how it can be produced for so small a sum. The frontispiece of the first number depicts a thrilling scene of adventure such as is delightful to boyish hearts. The story for which this serves as an illustration is headed, "For Glory and Renown," a story of the wars, by D. Parry. Smugglers, wolves, and subterranean passages are freely scattered over the narrative; little else need, therefore, be said in recommendation for the class for whom it is intended. There are articles on all manner of subjects of interest to boys, a "funny" page, and last, but not least, a series of prize schemes especially devoted to the encouragement of athletics. These are so arranged as to bring the possibility of winning a prize within the power of all sorts and conditions of boys, and even girls, who are remembered in this department of the paper, and will greatly add to the popularity of the new venture.

An amusing case came up at Bow Street the other day. A lady was summoned for keeping a male servant without a licence. Her defence was that the youth employed by her was practically a "housemaid," and did not therefore come under the Act. Whether the boy in question was content to personify one of the other sex history does not relate, but the magistrate would have none of it, and a fine was imposed.

---

The new Meath Home for Epileptics has been at work for about a week. Fifty out of the four hundred applicants have been received. Whilst the numbers who have been admitted into this haven have every reason to be thankful to Lady Meath and her many helpers, it must be a painful revelation to these kind hearts that so many are still in want of such a refuge. It will no doubt lead to the sphere of usefulness being rapidly extended, and to the same provision being made for male patients also. The £500 required for working expenses of the Home was so quickly collected that we can, with confidence, expect that all necessary funds for the continuance and extention of the excellent scheme will be also forthcoming.

---

We understand that Mr. G. Q. Roberts, M.A., Oxon., has taken up his residence at the London Hospital as House Governor and Secretary. It is not generally known that Mr. Roberts has for some years taken the most active interest in the welfare of waifs and strays, and that previous to his appointment as Secretary to the London he displayed commendable perseverance in qualifying himself for such a post. Since his appointment as Secretary he has done excellent service to the hospital by overhauling the estates and household property and introducing changes which have considerably improved the revenues. We are confident that the Committee and Governors have made a wise choice, and that Mr. Roberts will prove a most able and efficient successor to Mr. W. J. Nixon, of whose excellent services we have already given an account.

---

The Beverley Town Corporation has acted with admirable promptitude in rectifying the most important omission in their system, of isolation accommodation, which has caused the necessity for temporary measures on the approach of cholera. Whilst in so very many places, squabbles, indecision, and even blows are established instead of the necessary hospital, the Radcliffe Hospital tents which Beverley possesses are ready for occupation. They have been erected at a really small cost—less than £150. There are five separate departments, two tents with four beds in each, a matron's tent, mortuary tent, and galvanised iron kitchen, so that the group forms quite a little settlement. The tents are placed on asphalte and foot-paths have been laid. The marquee wards can be heated by hot water. The latrine arrangement will consist of troughs largely filled with tar. The Sanitary Committee are really to be congratulated on the success of their project, which was effected under the usual difficulties attendant on the choice of a site.

